# Impacts of human disturbance on ghost crab burrow morphology and distribution on sandy shores

**DOI:** 10.1371/journal.pone.0209977

**Published:** 2018-12-31

**Authors:** Mustafa R. Gül, Blaine D. Griffen

**Affiliations:** 1 Marine Science Program, School of the Earth, Ocean, and Environment, University of South Carolina, Columbia, South Carolina, United States of America; 2 Department of Biology, Brigham Young University, Provo, Utah, United States of America; University of Sydney, AUSTRALIA

## Abstract

Ghost crabs have been widely used as a bio-indicator species of human impacts on sandy beaches to obtain reliable biological data for management and conservation purposes. Ghost crab population densities and individual sizes decline dramatically under human pressure. However, distribution within a beach and the factors that determine this distribution of ghost crabs is still an open question. These factors may provide valuable information for understanding human impacts on sandy beaches. Here we examine ghost crab burrows on 20 sandy beaches of South Carolina, USA under various levels of human impacts to understand the response in terms of spatial distribution of this species to human impacts. We also examine the burrow characteristics and environmental properties of the burrows to determine whether these factors alter burrow characteristics. We show that crabs on heavily impacted beaches altered their spatial distribution to mostly occupy the edges of impacted beaches. Further, this change in spatial distribution was influenced by the size distribution of the population on a beach (i.e. larger individuals occupy upper parts on the beaches). We also found that ghost crabs altered the morphology of their burrows on heavily impacted beaches. Ghost crabs create deeper, steeper and smaller burrows under human impacts. These patterns were also influenced by physical characteristics of the beach. Our results suggest that human impacts can directly influence the spatial distribution of ghost crab populations within a beach and therefore sampling at upper parts of the beaches overestimates the population density and individual sizes. Our results support the use of ghost crabs as indicator species in effective beach management, but suggest that assessments would benefit from examining the morphology and distribution of burrows as opposed to simply using burrow counts to assess the health of sandy shores.

## Introduction

Human impacts have become the major force shaping ecosystems globally over the past century. The strength of these impacts in different regions are largely correlated with human population size and changes in the land-use dynamics [1]. Currently, human populations in coastal areas are growing faster than in other non-coastal regions [2].

Coastal ecosystems are highly preferred regions due to their valuable services [2–3] such as recreation and tourism [2,4]. While recreation and tourism provide a socio-economic boost [5–7], they cause important ecological issues [4] such as alteration in natural habitats, declines in biodiversity and increase in pollution [8], which can cause either reversible or irreversible damage in coastal ecosystems [3, 9].

The degree of ecological change in marine ecosystems is strongly correlated with the intensity of human impacts [8]. On sandy beaches, those ecological alterations mostly are changes in fauna and flora [9–11], decreases in abundance and biodiversity [6,9] and declining ecosystem services [12]. While human impacts may alter these aspects of marine ecosystems [13], the extent of human impacts is often monitored using bio-indicator species or assemblages [14]. Examples include bean clams, *Donax* spp. [15–17], mole crabs, *Emerita* spp. [18], sand beach coleoptera, *Phaleria* spp. [19] and ghost crabs, *Ocypode* spp. (see [20] and citations) and *Hoplocypode* spp. [21]. These species have been used extensively due to their high abundance and sensitivity to human impacts [22].

Ghost crabs have been widely used as a bio-indicator species to determine the ecological impacts of human use of sandy beaches globally ([20] and citations) because of their strong responses to anthropogenic [23] and natural impacts [24], as well as their relatively large size and characteristic behaviors [11]. Also, their burrowing behavior provides an advantage for a low-cost and efficient monitoring technique [25] that has been applied to many sandy beaches in various locations around the world such as in North Carolina [4, 24], Virginia and Maryland [10] Ghana [26, 27], Brazil [23], South Africa [28], as well as in Australia [11, 29–31].

While ghost crabs have been broadly used as indicator species, there are still aspects of their ecology and responses to human impacts that we do not understand. These include understanding the gradation in ghost crab responses, both in terms of individual behaviors and in terms of population abundance, across different levels of human impacts. Developing a better understanding of these factors will enhance the usefulness of ghost crabs as bioindicators of sandy beaches.

The purpose of this study was to investigate how various types and levels of human impacts alter the burrowing characteristics such as burrow volume, depth, inclination angle and shape and population levels like density, distribution and individual sizes of burrows of the Atlantic ghost crab, *Ocypode quadrata*, on South Carolina sandy beaches. We hypothesized that ghost crabs would produce smaller burrows and would be found in lower densities in areas that experienced the most extreme disturbance due to detrimental activities of visitors on sandy shores, and that burrowing properties would be more sensitive than burrow density as a bioindicator metric.

## Methods

### Study sites

**Fig 1:**
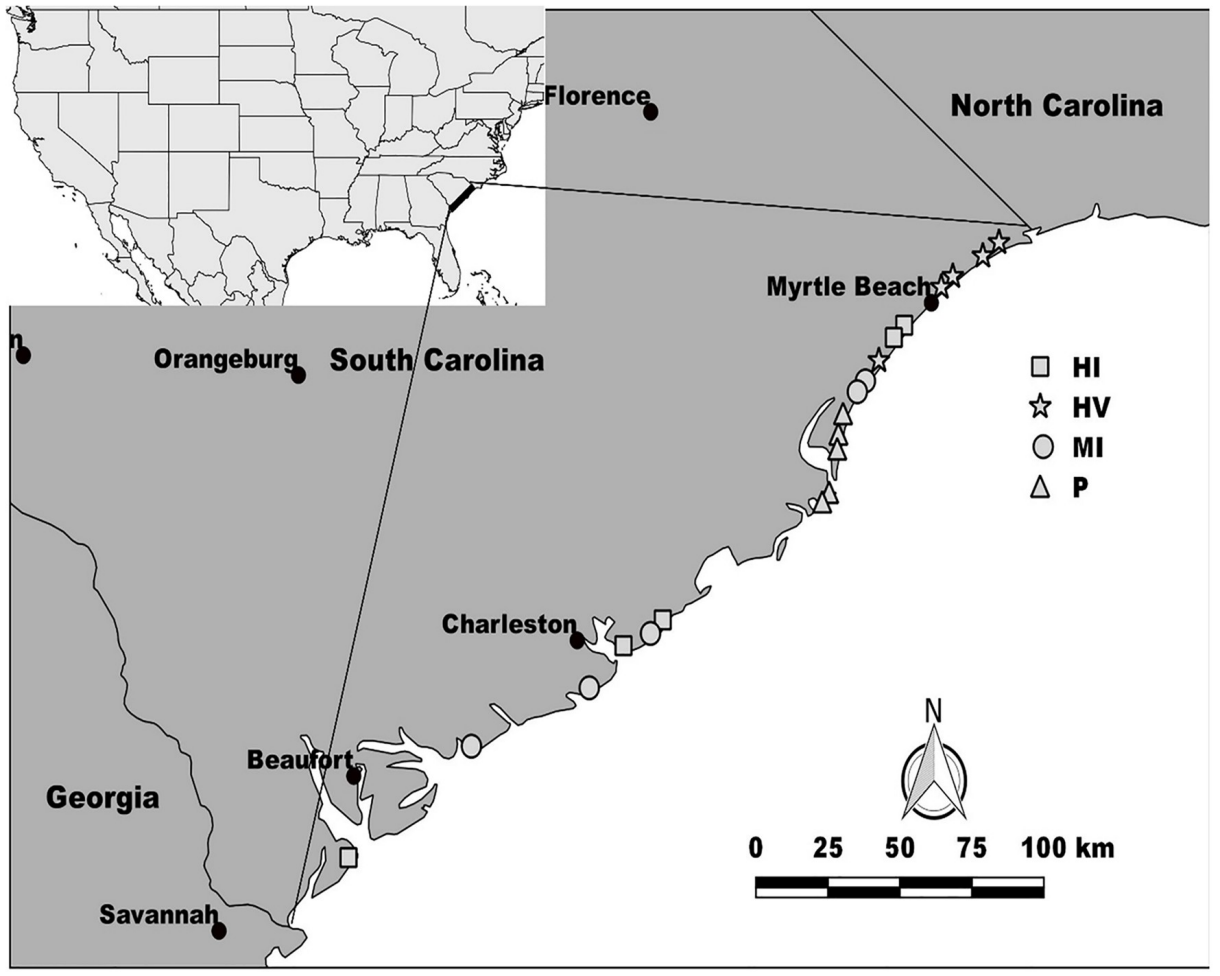
Study sites. In the map, P represents the pristine sites, MI represents the moderately impacted sites by only people, HI represents the highly impacted sites by only people and HV represents the highly impacted sites by people and vehicles. Map was created with QGIS v3.0 software.

Twenty sandy beaches under various levels of human disturbance located on the coast of South Carolina, United States were sampled between 26th May and 28th September 2016 (Fig 1). The total distance between the northernmost study site (North Myrtle Beach) and southernmost study site (Burkes Beach, Hilton Head) was about 300 km. All field research was conducted under a permit issued by South Carolina Department of Natural Resources (Permit Numbers: 4044, 4261). No protected species were disturbed during our research. All study sites are ocean exposed sandy beaches, however, because of a wide continental shelf, the wave energy affecting the coast of South Carolina is relatively low [32].

We divided our study sites in 4 different groups in terms of impact levels based on our observations of intensity of visitors, whether or not beaches experienced mechanical cleaning and presence or absence of the vehicles on the sand. In order to determine the intensity of the visitors on the beaches, we counted the number of people at each site using binoculars over a two-hour period from 0800 to 1000 h over approximately a linear kilometer of the beach during a normal workweek. We observed whether the beach was mechanically cleaned at night by noting both the vehicles and their tire-marks on the sand.

The sites that are not accessible by vehicles were considered as pristine sites, because no pedestrians or vehicles were observed. We considered the beaches as moderately impacted sites when they were visited by less than 50 pedestrians per hour. The number of visitors at moderately impacted sites ranged from 31 people h^-1^ at Pawley’s Island to 48 people h^-1^ at Folly Beach with an average of 39.3±17.5 (±S.D.) people h^-1^. On the other hand, the number of visitors at highly impacted sites by only people were between 54 people h^-1^ at Surfside Beach and 63.5 people h^-1^ at Sullivan’s Island with an average of 60.2±32.08 (±S.D.) people h^-1^. Finally, the visitor numbers at highly impacted sites by people and vehicles ranged from 58.5 people h^-1^ at Garden City Beach to 89.5 people h^-1^ at Myrtle Beach 1 with an average of 72.3±13.5 (±S.D.) people h^-1^. For a detailed description of the study sites see [33].

### Spatial burrow distribution

Gül and Griffen [33] used an indirect burrow examination technique on South Carolina sandy shores to understand whether ghost crab burrow density and size varied in relation to presence of human disturbance. They only examined burrows within 2.5 m of the backshore vegetation within each site and thus did not collect any data for spatial burrow distribution and the variation in the burrow density and size at different tidal heights.

In order to determine if the distribution patterns of the ghost crab burrows change due to human disturbance, we observed the burrow opening diameter (hereafter burrow diameter). At each site, we sampled in three replicate rectangular quadrats (10 m X 5 m) at each of five tidal heights parallel to the shore at low tides. Quadrats at the same tidal height within a beach were spaced ~100 m from each other. The first quadrat (T1) was deployed on the seaward site of the dune vegetation. The fourth (T4) and the fifth (T5) quadrats were applied on the landward and seaward sites of the strand line, respectively. Since the beach width varied among study sites, we positioned the second (T2) and third (T3) quadrats between T1 and T4 so that all were separated by approximately equal distances. This meant that the distance between quadrates T1-T4 differed across sites depending on the width of the shore.

In each quadrat, only the active ghost crab burrows were examined. We observed the fresh sand and/or traces left by individuals around the burrow mouths to decide if a burrow was active or not [29– 31]. Since crab size is positively related to its burrow opening diameter in Atlantic ghost crab (r^2^ = 0.98; [34]), we examined ghost crab burrows by measuring the largest distance of the burrow openings as burrow diameter using a Vernier caliper (±0.1 mm) and the distance between the burrow openings and the backshore vegetation (hereafter distance). When the burrow had more than one opening, we considered only the main opening by observing the traces around it. We used a linear mixed effects model to determine whether the size of ghost crab burrows varied among tidal heights and the beaches that were under various levels of human disturbance. Human impact level was treated as categorical fixed factor. Human impact types and quadrats (i.e. tidal height on the beach) were treated as categorical and ordinal fixed factors, respectively. To control for daily and latitudinal difference, sampling time as julian day and latitude of the sampling site were added as random factors to this analysis and all subsequent statistical analysis in this paper. Also, for this and all subsequent statistical analysis, we used Tukey’s multiple comparison test to determine the pairwise differences among impact types. We used the statistical software R version 3.3.2 [35] for all statistical analyses.

To understand if the burrow density (burrows/m^2^) was affected in different parts of beaches under various impact levels, we counted the number of the active burrows in each quadrat and analyzed these data using a generalized linear mixed effects model (GLMM) with a Poisson distribution. The impact level was treated as fixed factor and the quadrat number (i.e., relative height on the shore) was treated as an ordinal variable.

### Burrow architecture

The following procedure was conducted after sunset to make sure that all individuals had already left their burrows. Burrows (1 to 3 burrows from each quadrat level with various opening sizes (except T5 because it was often submerged) were selected randomly. We poured a 2:1 mixture of plaster of Paris and freshwater into the burrows until they were fully filled [36]. We waited for about 30 minutes until the plaster was dried, after which we excavated the casts using a shovel. We measured the angle of inclination of the casts relative to horizontal. After excavation, we measured the depths using a ruler from the surface to the deepest point of the casts. We then labeled the casts and took them to the laboratory for further investigations.

In the laboratory, we measured the diameter every three-centimeters along the length of each cast using a Vernier caliper (±0.1 mm) and obtained the average diameter of each cast. The volume of the casts was obtained by using the equation for the volume of cylinder and using the average diameter of each cast. The shapes and the patterns of the casts were obtained by visual observations. We used chi-square to test the variation in burrow shapes between human impact levels.

Burrow size and morphology may also be influenced by physical characteristics of the site. Thus, we measured the surface temperature and moisture (i.e. relative humidity) for 10 minutes every hour during the night using a data logger (Onset U23-001, HOBO Pro v2). We also measured the temperature and moisture—of the burrows at the deepest point just after we excavated the casts -. Because these excavations were done at night, we assume that the brief aerial exposure following excavation and before measurement did not substantially influence these measurements; and any influence on temperature or moisture from aerial exposure that did occur would have been similar across all burrows, and therefore would not influence our cross-site comparison.

To obtain geomorphological properties of the study sites, we measured the sand compaction, sand grain size and beach slope. We obtained the sand compaction by using a pocket penetrometer (AMS G281) applied five times in each site. Since the strength of the sand was very low, we applied the penetrometer with its adaptor foot that increases the surface area of the measurement by 16 times. At the end of the measurements, we divided our results by 16 to obtain the actual compaction values. For grain size, we used the sieving method [37]. We collected three 500 g samples from each site. The sand was dried and separated through a series of sieves with different mesh sizes (2 mm, 1 mm, 0.5 mm, 0.42 mm, 0.25 mm, 0.177 mm, 0.125 mm and 0.074 mm). Finally, we obtained the slope of the study sites by dividing the elevation difference between the beginning of the backshore vegetation and the strandline by the distance between those two points. For each study site, the same technique was applied three times.

Since the burrow volume and depth are positively correlated with the size of burrow opening [36, 38], which itself is a proxy of crab size [34], we regressed log-transformed burrow architectural data against log-transformed burrow diameter to remove the effects of crab size [39]. The standardized residuals from those regressions were used as response variables for all subsequent statistical analyses for burrow architecture. We used linear mixed effects models to examine the factors that influenced the variation in burrow metrics. The residuals of burrow volume and depth, after accounting for crab size, and inclination angle were treated as the response variables in separate analyses. The suite of predictor variables varied depending on the expected relationships for each response variable. For the model examining burrow volume, we used impact level, sand grain size, distance from backshore vegetation, sand compaction and all the possible interactions of those variables as fixed factors. For the model examining burrow depth, we used burrow temperature and humidity in addition to the variables listed above. Random factors were the standard factors described above for all analyses in this study. For each of these analyses, we selected the best fitting models using the lowest AICc values (*dredge* function in MuMIn package in R, [40]). When more than one model with ΔAICc < 2 existed, the models were averaged [41].

## Results

### Burrow size and distance from backshore vegetation

**Fig 2:**
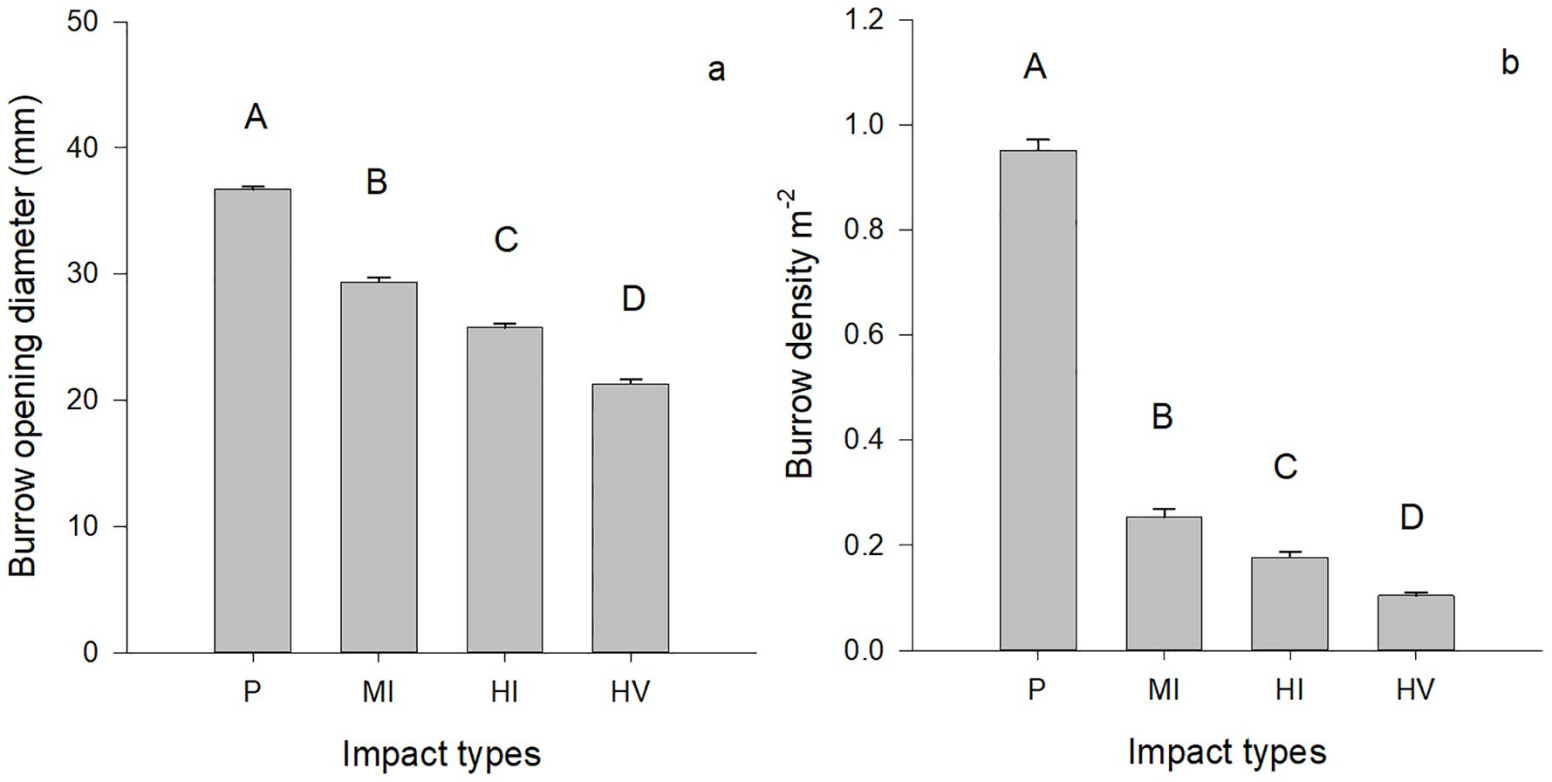
Overall ghost crab burrow density and opening diameter among impact types. Variation in burrow density (mean burrow/m^-2^ ±S.E.) (a) and burrow opening diameter (mm ±S.E.) (b) for *O*. *quadrata* among impact types on South Carolina beaches. (P = pristine sites, MI = moderately impacted sites, HI = highly impacted sites by only people, HV = highly impacted sites by people and vehicles). Letters above bars represent significant differences.

A total of 5565 ghost crab burrows were examined in terms of their size, density and the distance from vegetation. Burrow diameter showed a notable difference in the sites that were heavily disturbed compared to less impacted sites (Fig 2). The burrow diameter in pristine sites was significantly higher than the burrow diameter in the sites that were moderately impacted (estimate = -7.39, z = -7.11, p < 0.001), highly impacted by people (estimate = -10.74, z = -10.03, p < 0.001) and highly impacted sites by people and vehicles (estimate = -15.26, z = -13.33, p < 0.001). Unsurprisingly, a higher average of burrow diameter was found in moderately impacted sites compared to highly impacted sites by people (estimate = -3.35, z = -3.28, p = 0.005) and highly impacted sites by people and vehicles (estimate = -7.87, z = -7.15, p <0.001). Also, presence of vehicles on the sandy shores caused a lower burrow diameter in the sites that are heavily disturbed (estimate = -4.51, z = -3.39, p < 0.001, Fig 2).

**Fig 3:**
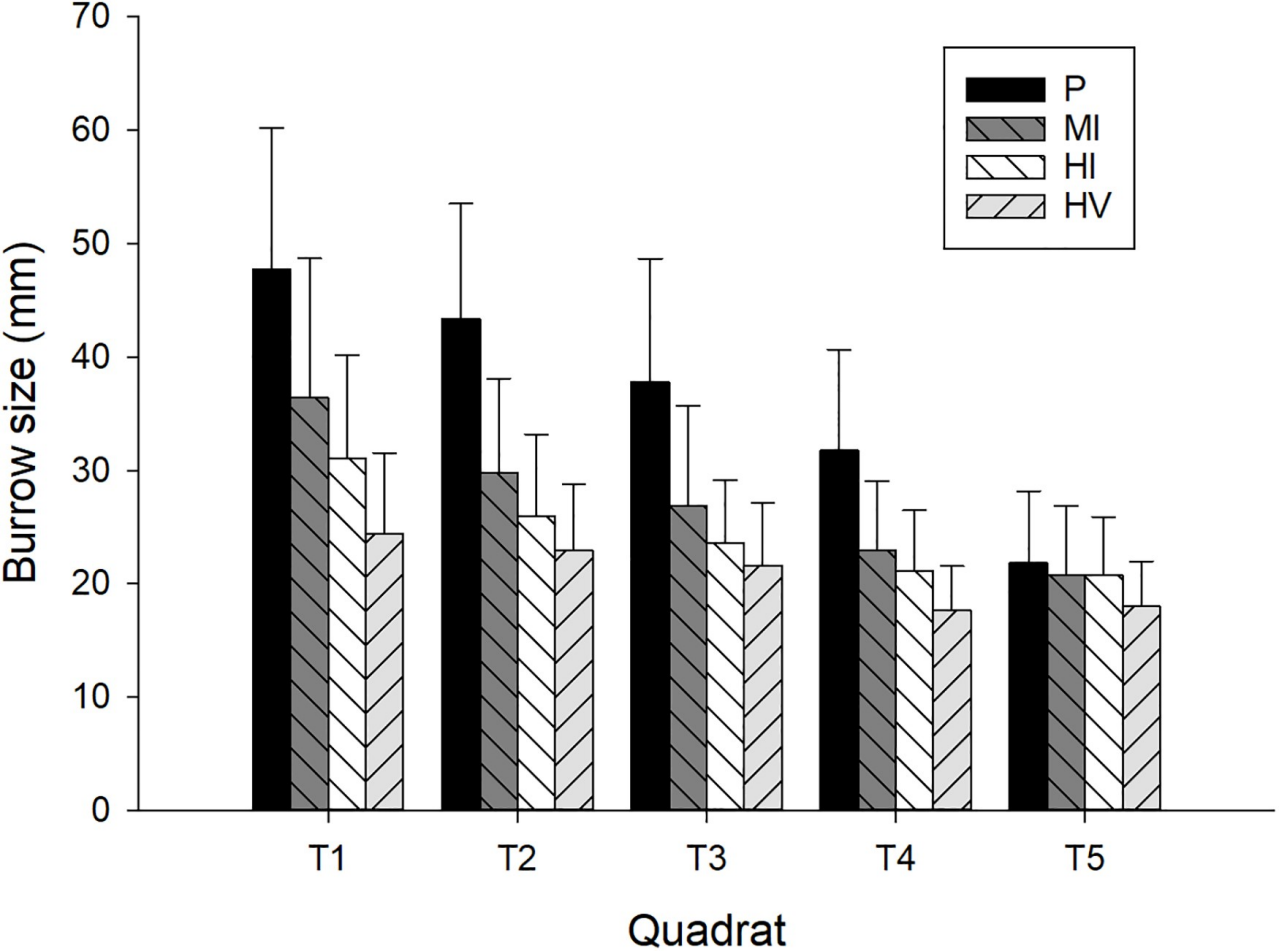
Ghost crab burrow diameters among impact types. Relationship between the diameter (mm) of *O*. *quadrata* burrows and the distance from backshore vegetation (m) on South Carolina sandy beaches (a = pristine sites, b = moderately impacted sites, c = highly impacted sites by only people, d = highly impacted sites by people and vehicles).

We found zonation in burrow size and location on the beaches in all disturbance levels (Fig 3). The results of LME indicated that there was clear difference in the zonation of burrow sizes in pristine sites compared to moderately impacted sites (LME, estimate = 2.53, t = 11.16, p < 0.001) and highly impacted sites (by people: LME, estimate = 2.53, t = 14.29, p < 0.001 and by people and vehicles: LME, estimate 4.71, t = 14.78, p < 0.001). Random factors were as follows: latitude variance component = 0.275 (S.E. = 0.524) and Julian days variance component = 1.013 (S.E. = 1.006).

### Burrow density

**Fig 4:**
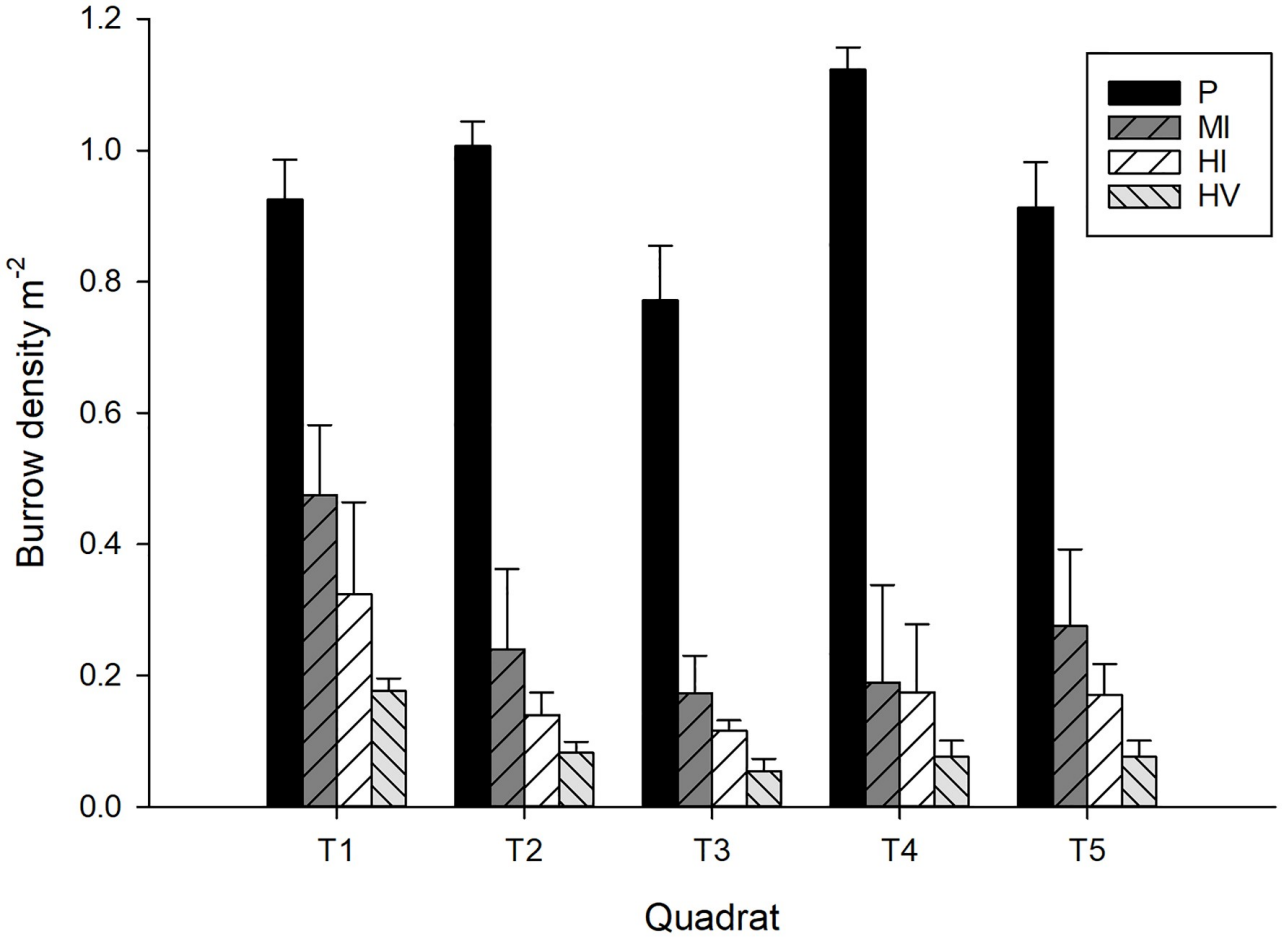
Ghost crab burrow density among impact types and height of the beaches. *O*. *quadrata* burrow density (burrows/m^2^ ± S.D.) on South Carolina sandy beaches with various disturbance levels and with height on the shore (transects).

We found that the zonation of ghost crab burrows was altered by human impacts. While, the burrow density across beaches in pristine sites were similar to each other, ghost crabs mostly occupied the edges of the sites (upper and lower parts of beaches) under human impacts. The ghost crab burrow density was significantly lower in the sites that were under human impacts compared to pristine sites, and also differed among quadrats (Table 1, Fig 4). Expectedly, burrow density was lower in moderately impacted (estimate = -0.68, z = -5.13, p < 0.001), highly impacted by people (estimate = -1.15, z = -8, p < 0.001) and highly impacted by people and vehicles (estimate = -1.88, z = -11.6, p < 0.001) sites compared to the burrow density in pristine sites. Both highly impacted sites by people (estimate = -0.47, z = -3.27, p = 0.0058) and highly impacted sites by people and vehicles (estimate = -1.2, z = -7.39, p < 0.001) had lower burrow densities than moderately impacted sites. Additionally, vehicle impacts on sandy shores caused a lower burrow density (highly impacted sites by people vs highly impacted sites by people and vehicles: estimate = -0.73, z = -4.3, p <0.001, Fig 2).

**Table 1:** Summary of generalized linear mixed effects model (GLMM) with poisson distribution showing the burrow density (burrow/m^2^) in quadrats and sites under various types of human impacts compared to pristine sites.

**fixed factor**	**S.E.**	***Z*-value**	***p*-value**
*A* (moderate impact)	0.136	-5.01	<0.001*
*B* (high impact by only people)	0.144	-8.01	<0.001*
*C* (high impact by people and vehicle)	0.162	-11.6	<0.001*
*D* (quadrat)	0.011	0.67	0.49
*A* x *D*	0.026	-8.89	<0.001*
*B* x *D*	0.030	-6.28	<0.001*
*C* x D	0.037	-3.03	<0.001*
**Random factor**	Variance component		S.E.
*Latitude*	<0.001		<0.001
*Julian days*	0.022		0.151

* indicates the significant values

### Burrow architecture and metrics

**Fig 5:**
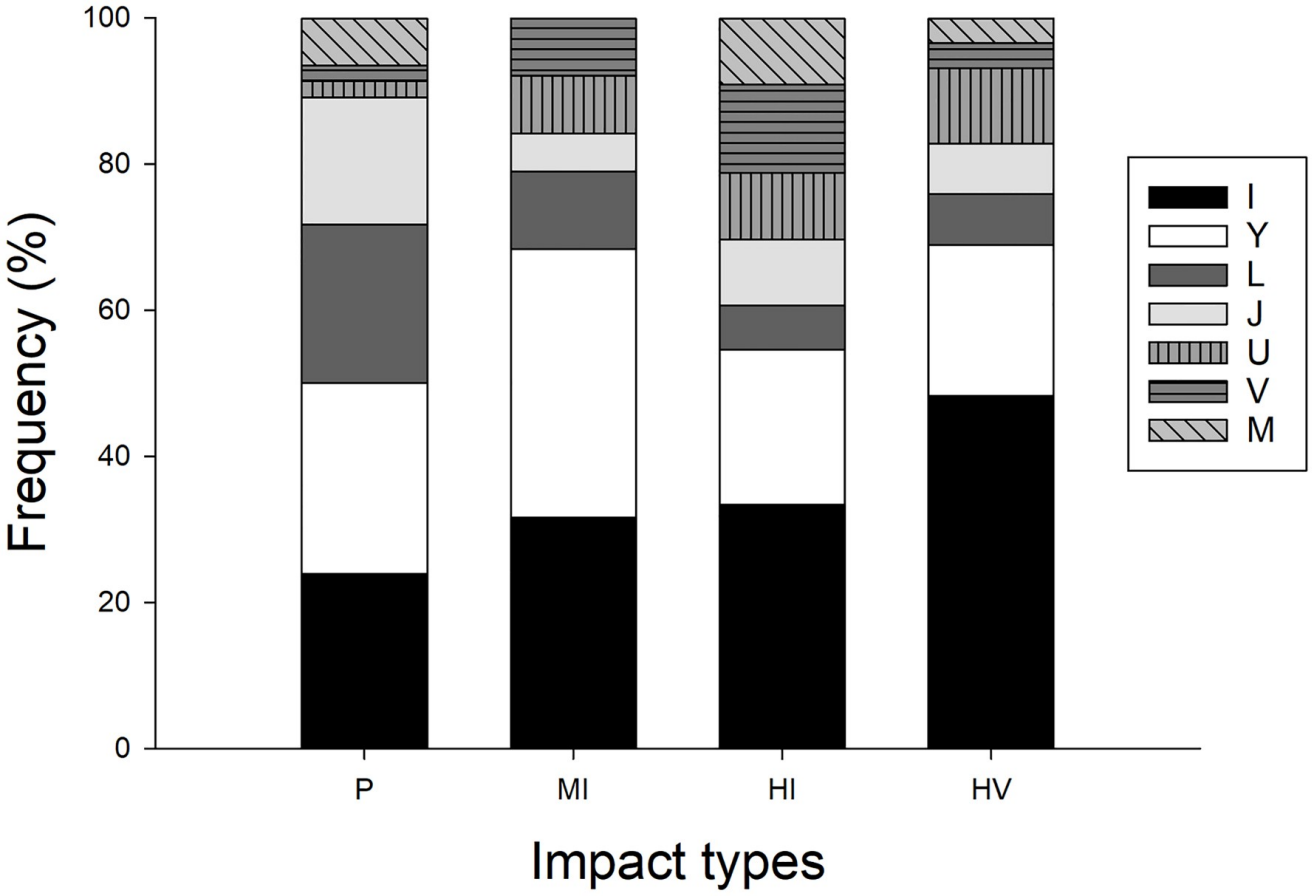
Variation in burrow shapes among impact types. Percent frequency (%) of *O*. *quadrata* burrow shapes among impact types on South Carolina beaches.

We examined a total 146 ghost crab burrow casts of seven shapes resembling the letters I, Y, L, J, U, V and M. Regardless of impact level, most of the burrows were of I (n = 47) with 32.87% and Y (n = 39) with 26.71% shapes (Fig 5). Burrows with single-opening shapes (I, J, L) were more common (55.47%) than more complex-shaped with multi-openings burrows (Y, U, V, M) (44.52%). Burrow shapes in pristine sites differed significantly compared to burrow shapes in moderately impacted sites (*Chi-squared* test, *X*2 = 418.4, df = 42, p < 0.001) and in heavily impacted sites (by people: *Chi-squared* test, *X*2 = 438, df = 49, p < 0.001 and by people and vehicles: *Chi-squared* test, *X*2 = 303.26, df = 49, p < 0.001). In addition, burrow shapes in moderately impacted sites were different from the burrow shapes in highly impacted sites by people (*Chi-squared* test, *X*2 = 531.33, df = 42, p < 0.001) and in highly impacted sites by people and vehicles (*Chi-squared* test, *X*2 = 330.54, df = 42, p < 0.001). Burrow shapes also changed with the impact of vehicles in heavily impacted sites (*Chi-squared* test, *X*2 = 527.53, df = 49, p < 0.001). Unlike the previous studies on different ghost crab species [31], we found no chambers at any parts of the burrows.

**Fig 6:**
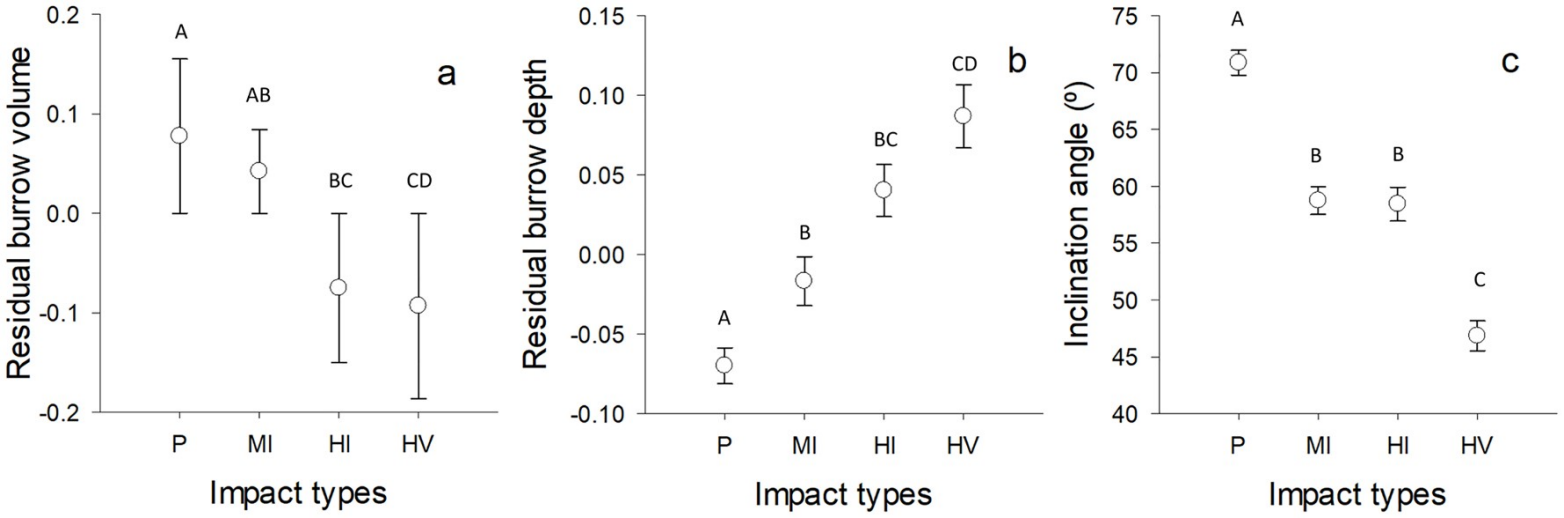
Burrow metrics among impact types. Variation in residual burrow volume (mean ± S.E.) (a), residual burrow depth (mean ± S.E.) (b) and inclination angle (mean ± S.E.) for *O*. *quadrata* among impact types on South Carolina beaches. Capital letters within graph represent significant differences (c).

Metrics of the burrow casts clearly varied among sites based on impact level. Specifically, ghost crab burrow volume was similar between heavy impacted beaches (highly impacted site by people vs highly impacted sites by people and vehicles: z = -1.03, p = 0.69) and also between slightly impacted beaches (moderately impacted sites vs pristine sites: z = -2.4, p = 0.065). Additionally, ghost crabs appeared to create larger burrows in pristine sites compared to highly impacted sites by people (z = -3.12, p = 0.008) and highly impacted sites by people and vehicles (, z = -3.27, p = 0.004). Surprisingly, there was no significant difference between moderately impacted sites and highly impacted sites by people (, z = -2.32, p = 0.08) in terms of burrow volume (Table 2, Fig 6). According to the best fitting models, geomorphological properties of the sandy beaches (i.e. sand compaction and grain size) were correlated with the burrow volume (Table 2). The average sand compaction (range: 0.04–0.131 kg cm^-2^) and grain size (range: 0.238–0.52 mm) were 0.086 kg cm^-2^ and 0.32 mm, respectively.

**Table 2:** Summary of linear mixed effects model (LMER) with a Gaussian distribution showing the residual burrow metrics (burrow volume (cm^3^), burrow depth (cm) and burrow inclination angle (°) of ghost crab populations after accounting for crab size in the sites under various types of human impacts compared to pristine sites.

	**Burrow volume**		
**fixed factor**	**S.E.**	***t*-value**	***p*-value**
***A* (moderate impact)**	0.075	-2.4	0.029*
***B* (high impact by only people)**	0.094	-3.12	0.006*
***C* (high impact by people and vehicle)**	0.104	-3.27	0.004*
***D* (compaction)**	6.142	2	0.063
***E* (grain size)**	1.875	2.21	0.04*
***D x E* (grain size x compaction)**	18.181	-1.95	0.067
**Random factor**	Variance component		S.E
***Julian days***	<0.001		<0.001
***Latitude***	<0.001		<0.001
	**Burrow depth**		
***A***	0.028	2.12	0.033*
***B***	0.03	3.79	0.0001*
***C***	0.029	5.086	<0.0001*
***F* (humidity)**	0.057	0.002	0.99
***G* (temperature)**	0.047	2.23	0.025*
***F x G***	0.021	0.002	0.99
**Random factor**	Variance component		S.E
*Julian days*	0.0005		0.023
*Latitude*	0.0045		0.094
	**Inclination angle**		
***A***	2.72	-4.87	0.0008*
***B***	2.65	-5.04	0.0006*
***C***	2.75	-9.15	<0.0001*
***E***	14.8	0.94	0.36
***H*** **(distance)**	0.104	-1.57	0.11
**Random factor**	Variance component		S.E
*Julian days*	<0.001		<0.001
*Latitude*	7.306		2.703

* indicates the significant values

**Fig 7:**
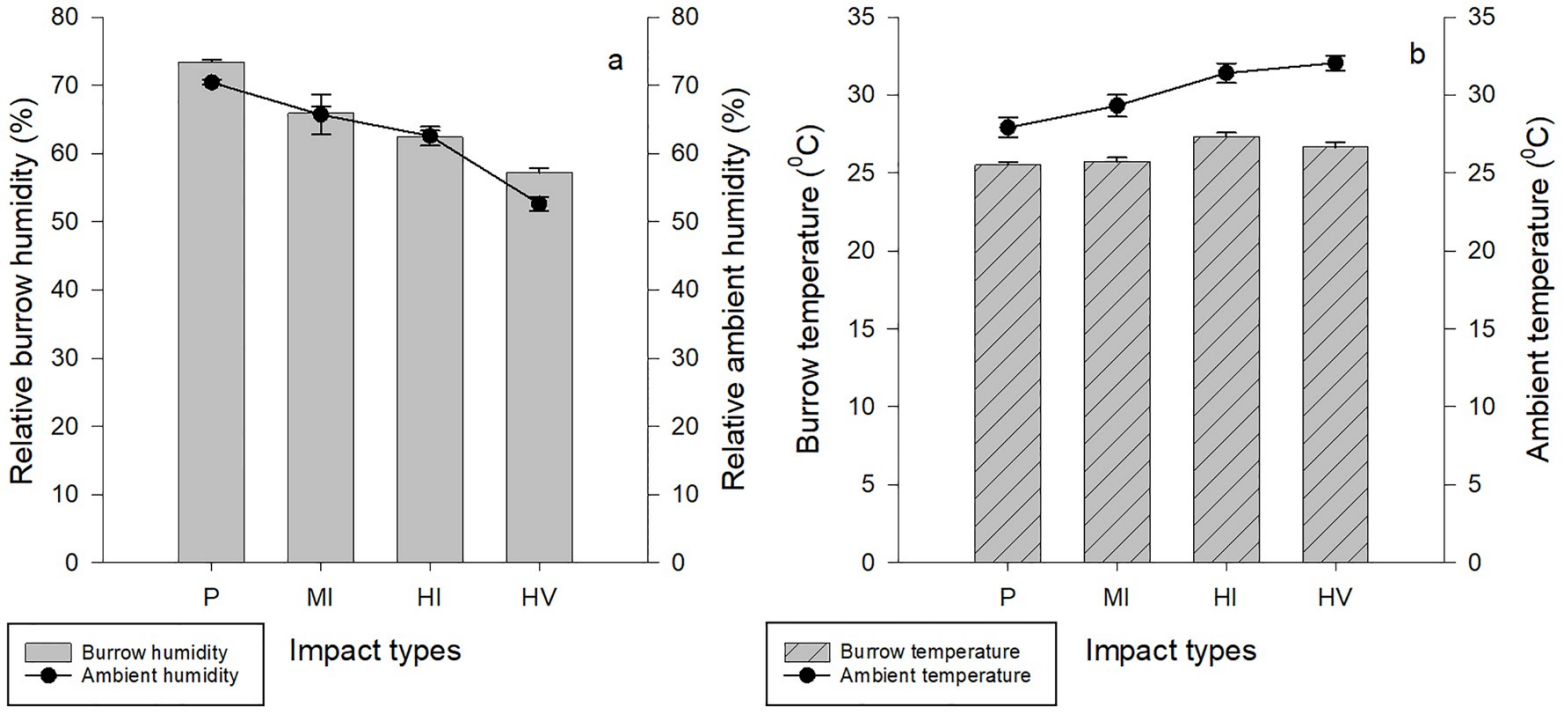
Environmental properties. Variation in environmental properties in *O*. *quadrata* burrows and among disturbance types. a) relative ambient and burrows humidity (mean ± S.E.) among disturbance types, b) burrow and ambient temperature (mean ± S.E.) among disturbance types.

Ghost crabs appeared to dig deeper burrows as the level of human impact increased. Ghost crab burrows were shallower in pristine sites compared to moderately impacted sites (z = 1.87, p = 0.023), highly impacted sites by people (z = 3.15, p = 0.008) and highly impacted sites by people and vehicles (z = 3.92, p < 0.001). However, burrow depths were similar in moderately impacted and highly impacted sites by people (z = 1.66, p = 0.33), the depth was higher in highly impacted sites by people and vehicles (z = 2.72, p = 0.032). Also, there was no difference in burrow depth between highly impacted sites by only people and by people and vehicles together (z = 1.12, p = 0.67, Table 2, Fig 6). Moreover, inclination angle became steeper with increasing human impact and sand grain size and the distance from the backshore vegetation. Ghost crab burrows were steeper in highly impacted sites by people and vehicles compared to burrows in pristine (z = -9.15, p < 0.001), moderately impacted sites (z = -4.81, p < 0.001) and highly impacted sites by people (z = -4.68, p < 0.001). The inclination angle of the ghost burrows was lower in both moderately impacted (z = -4.87, p <0.001) and highly impacted sites by people (z = -5.04, p <0.001) compared the burrows in pristine sites (Table 2, Fig 6The best fitting model showed that burrow inside temperature was correlated with burrow depth, but relative humidity was not (Table 2, Fig 7).

## Discussion

We have shown that the distribution of the *O*. *quadarata* burrows on sandy beaches vary among the sites that are under influence of various levels and the types of human disturbance. We have further demonstrated that *O*. *quadrata* populations responds strongly to various human impact types by declining in both size and density, and burrowing characteristics and burrow dimensions vary based on human impacts on sandy beaches.

Previous studies have shown that environmental variables can also influence ghost crab burrow density, size and architecture such as temperature, moisture level, wind [31], and geo-morphological properties of the sandy shores like sand compaction, beach slope and sand grain size [42– 43] and as well as erosion [24]. Since the main goal of this work was to understand the impact of human disturbance, we did not examine these other factors.

### Burrow density and size

The results of our study reflect that the overall density and the average burrow size dramatically decline at the sites where human impacts exist. Several studies have shown that vehicles [4, 7, 10, 16, 24, 28– 31], intense pedestrian trampling [23,29] and cold fronts [44] cause a significant decline in number and size in ghost crab populations and our findings in terms of reductions in ghost crab populations are consistent with those studies. It has been found that individuals using burrows that are shallower than 25 cm are likely to be crushed by vehicles [6]. This finding may explain the reason why the density is low at the sites where vehicles are allowed, especially when they are allowed during the day time when crabs are in their burrows. It seems reasonable to explain the decline in the individual crab size under vehicles disturbance by considering the direct crushing by vehicles during night times [4], since the larger individuals are slower than the middle size ones [45–46] and so they may not be fast enough to escape vehicles during night time activity. But, these arguments cannot explain the decline in the ghost crab populations in the sites where the vehicles are not present. During our sampling, we observed people catching and playing with crabs during night times. They mostly catch the larger individuals, possibly because they are easier to see and catch. Stress from this type of human encounter may contribute to the decline in density and size for those sites that have intense human impact.

Changes in food abundance may also contribute to ghost crab declines. Common prey items including bean clams (*Donax* spp.), mole crabs (*Emerita* spp.) and sandy beach coleoptera (*Phaleria* spp.) decline dramatically due to human disturbance [16– 19]. This decline in food items may alter energy dynamics of ghost crabs in ways that limit growth rate. Reductions in bean clams and mole crabs may be especially problematic since they are the most preferred food items given their high caloric content [34]. If this is the case, then smaller individuals in highly disturbed sites may potentially be similar ages as larger individuals in pristine and moderately impacted sites, reflecting lower growth rates due to food limitation. Overall, the reason for the reductions in the density and the individual size of ghost crab populations is still not clear [6] and most probably cannot be explained by using a single mechanism. Further research is required to investigate the possible reductions in the growth rate of ghost crabs related to food availability in highly disturbed sites.

Additionally, it is possible that the number of burrows (i.e. territory size) differs for crabs on beaches with different levels of human impact [47]. If so, burrow counts could cause spurious estimates of crab density across beaches. But, it should not change the overall results of the burrow size, since individuals excavate burrows relative their carapace width [34]. Moreover, a higher mortality or injury rate for larger individuals on the drift zones in unprotected beaches due to cold fronts may contribute to the lower density [44].

### Spatial distribution of ghost crab burrows

Our findings demonstrate that the burrow density is relatively homogenous across the pristine and moderately impacted beaches, but that individuals mostly prefer the edges of the beaches for burrowing in highly disturbed sites. Also, smaller individuals mostly occupy the landward areas of the strandline and the upper intertidal areas in highly disturbed sites. Contrary to this, smaller individuals (even juveniles) are more evenly dispersed across the shore in pristine and moderately impacted sites. Our findings in terms of lower moisture content at the sites where a high level of human impact exists are consistent with [10] and [7] probably due to the high compaction rate. As semi-terrestrial organisms, ghost crabs can burrow in both dry and wet areas [48] but always need to moisten their gill chambers for respiration [49]. Also, the frequency of moistening their gill chambers varies based on size (i.e. juveniles require more frequent replenishment of the water in their gill chamber than adults) [49]. Thus, finding the smaller burrows next to the strandline seems reasonable, since smaller individuals are not capable of digging as deep burrows [36] to access the sufficient moisture content in the sand [49]. Also, reductions in the inclination angle and the increase in size-independent burrow depth with increasing impact level might be evidence for the burrowing behavior of accessing an area of higher moisture content. While these arguments seem reasonable, they do not explain the fact that juveniles were found dispersed across all portions of pristine beaches. This finding could therefore indicate that juveniles are not required to renew the water in their gill chamber as often as anticipated, or that they spend more time outside of their burrows and access water during day time [49] in pristine and moderately impacted sites.

Biological reasons could also explain the distribution patterns of the individuals in various sizes. According to our findings here, in highly impacted sites, mostly smaller individuals occupy the areas closer to strandline and larger individuals occupy the areas closer to dunes that are more stable parts of the beaches. The higher density next to the backshore vegetation may be due to less impact by cleaners especially in the beaches that are mechanically cleaned. We have observed that the cleaners do not approach the vegetation, leading to less frequent disturbance in that part of the beach. Thus, the distribution may reflect the presence of vegetation. It seems reasonable to conclude that larger individuals competitively exclude smaller ones from those more stable portions of the beach. More studies are required to investigate the biological influences on the distribution of the ghost crabs under various impact levels and types such as intraspecific competition for space.

Ghost crabs are relatively intolerant to cold temperatures because of the associated low humidity [50]. However, while the moisture pattern of the ghost crab burrows has been studied, it remains unclear whether burrow temperature plays any role on the cross-shore distribution of ghost crab burrows [51]. Our results show that *O*. *quadrata* burrow characteristics are correlated with burrow moisture content, suggesting that burrow humidity may be an important factor. In highly disturbed sites, crabs dig deeper burrows, perhaps to reach more humid sediments. We have also shown that the temperature inside of *O*. *quadrata* burrows is not as important as moisture level for determining burrow morphology. These findings suggest that regardless of life stage and the position on the beach, ghost crabs require similar amount of moisture and they can tolerate variations in temperate in their burrows.

### Burrow architecture and metrics

We expected to find simpler burrow construction in highly disturbed sites especially when the site is impacted by the vehicles because of higher energy requirements of the complex-shaped burrows [7]. We instead found that *O*. *quadrata* create similarly shaped burrows under all types and levels of human impacts, although the proportion of the complex-shaped (Y, U, V, M) burrows varied among the level of human impacts. Higher proportion of the burrows with second arm could be a strategy of escaping from the potential predators or water entry from wave splash [52]. Since we have found no relation between burrow shapes and location within a beach, it is more likely that individuals create those complex-shaped burrows as a precaution against predators, or cannibals, rather than water entry.

Our finding of simple burrow construction is consistent with previous reports for this species [53],and may be an energy saving strategy [7]. The smaller and deeper burrows in highly impacted sites by people and vehicles support this idea. Physical pressure exerted by the weight of vehicles is greater than that produced by pedestrians and thus should reach deeper points. Thus, individuals excavate deeper, smaller and simpler burrows at highly impacted sites by people and vehicles both to save energy while still attaining protection and reaching the appropriate moisture.

## Conclusion

We demonstrate that *O*. *quadrata* alters the density, individual size, burrowing behavior and type and distribution patterns with increasing disturbance on sandy shores. These strong responses to even a small change in human disturbance makes *O*. *quadrata* a highly reliable bio-indicator species to determine and monitor the ecological consequences of human impacts on sandy beaches. Coastal managers often require ecological data to set up successful conservation plans. As a simple tool, burrow counts have been used previously (*see* [20]) as a reliable indicator of population density and dynamics and individual sizes [34, 54–56]. As mentioned by [57] for density of sandy beach organisms, we suggest cross-shore sampling to determine the variations in population structure, burrowing behavior and distribution pattern, all of which can increase the strength of the data collected for management purposes. We further suggest that combining the abundance and morphology data of ghost crab burrows for determining the health of sandy shores is a promising and low-cost technique for coastal managers.

## References

[1] Ojima DS, Galvin KA, Turner BL II. The global impact of land-use change. BioScience. 1994; 44(5): 300–304. 10.2307/1312379

[2] Davenport J, Davenport JL. The impact of tourism and personal leisure transport on coastal environments: a review. Estuar Coast Shelf Sci. 2006; 67(1–2): 280–292. 10.1016/j.ecss.2005.11.026

[3] McLachlan A, Brown A. The ecology of sandy shores. Elsevier; 2006.

[4] Wolcott TG, Wolcott DL. Impact of off-road vehicles on macroinvertebrates on a Mid-Atlantic beach. Biol Conserv.1984; 29(3): 217–240. 10.1016/0006-3207(84)90100-9

[5] James RD. From beaches to beach environments: linking the ecology, human-use and management of beaches in Australia. Ocean Coast Manage. 2000; 43: 495–514.

[6] Schlacher TA, Thompson L, Price S. Vehicles versus conservation of invertebrates on sandy beaches: mortalities inflicted by off-road vehicles on ghost crabs. Mar Ecol. 2007; 28(3): 354–367. 10.1111/j.1439-0485.2007.00156.x

[7] Lucrezi S, Schlacher TA. Impacts of off-road vehicles (ORVs)on burrow architecture of ghost crabs (Genus *Ocypode*) on sandy beaches. Environ Manege. 2010; 45(6): 1352–1362. 10.1007/s00267-010-9491-5 20411260

[8] Halpern BS, Walbridge S, Selkoe KA, Kappel CV, Micheli F, D’Agros C, et al A global map of human impact on marine ecosystems. Science. 2008; 319: 948–952. 10.1126/science.1149345 18276889

[9] Defeo O, McLachlan A, Schoeman DS, Schlacher TA, Dugan J, Jones A, et al Threats to sand beach ecosystems: a review. Estuar Coast Shelf Sci. 2009; 81(1): 1–12. 10.1016/j.ecss.2008.09.022

[10] Steiner AJ, Leatherman SP. Recreational impacts on the distribution of ghost crab *Ocypode quadrata* Fab. Biol Conserv. 1981; 20: 111–122.

[11] Barros F. Ghost crabs as a tool for rapid assessment of human impacts on exposed sandy beaches. Biol Conserv. 2001; 97(3): 399–404. 10.1016/S0006-3207(00)00116-6

[12] Barbier EB, Hacker SD, Kennedy C, Koch EW, Stier AC, Silliman BR. The value of estuarine and coastal ecosystem services. Ecol Monogr. 2011; 81(2):169–193. 10.1890/10-1510.1

[13] Vitousek PM, Mooney HA, Lubchenco J, Melillo JM. Human domination of Earth’s ecosystems. Science. 1997; 277: 494–499. 10.1126/science.277.5325.494

[14] Solomon M, Van Jaarsveld AS, Biggs HC, Knight MH. Conservation targets for viable species assemblages? Biodivers Conserv. 2003; 12: 2435–2441. 10.1023/A:1025805731366

[15] Defeo O, de Alava A. Effects of human activities on long-term trends in sandy beach populations: the wedge clam *Donax hanleyanus* in Uruguay. Mar Ecol Prog Ser. 123: 72–82.

[16] Schlacher TA, Thompson LMC, Walker SJ. Mortalities cause by off-road vehicles (ORVs) to a key member of sandy beach assemblages, the surf clam *Donax deltoids*. Hydrobiologia. 2008; 610: 345–350. 10.1007/s10750-008-9426-9

[17] Sheppard N, Pitt KA, Schlacher TA. Sub-lethal effects of off-road vehicles (ORVs) on surf clams on sandy beaches. J Exp Mar Biol Ecol. 2008; 380: 113–118. 10.1016/j.jembe.2009.09.009

[18] Cardoso RS, Barboza CAM, Skinner VB, Cabrini TMB. Crustaceans as ecological indicators of metropolitan sandy beach health. Ecol Indic. 2016; 62: 154–162. 10.1016/j.ecolind.2015.11.039

[19] González SA, Yáñez-Navea K, Muñoz M. Effect of coastal urbanization on sandy beach coleoptera *Phaleria maculate* (Kulzer, 1959) in northern Chile. Mar Pollut Bull. 2014; 83(1): 265–274. 10.1016/j.marpolbul.2014.03.042 24768173

[20] Schlacher TA, Lucrezi S, Connoly RM, Peterson CH, Gilby BL, Maslo B, et al Human threats to sandy beaches: A meta-analysis of ghost crabs illustrates global anthropogenic impacts. Estuar Coast Shelf Sci. 2016; 169: 56–73. 10.1016/j.ecss.2015.11.025

[21] Sakai K, Türkay M. Revision of the genus *Ocypode* with the description of a new genus, *Hoplocypode* (Crustacea: Decapoda: Brachyura). Memoirs of the Queensland Museum. 2016; 56(2): 665–793.

[22] Carignan V, Villard MA. Selecting indicator species to monitor ecological integrity: a review. Environ Monit Assess. 2002; 78(1): 45–61. 12197640 10.1023/a:1016136723584

[23] Neves FM, Bemvenuti CE. The ghost crab *Ocypode quadrata* (Fabricius, 1787) as a potential indicator of anthropogenic impact along the Rio Grande do Sul coast, Brazil. Ecol Indic. 2006; 133(4): 431–435. 10.1016/j.biocon.2006.04.041

[24] Hobbs CH, Landry CB, Perry JE. Assessing anthropogenic and natural impacts on ghost crabs (*Ocypode quadrata*) at Cape Hatteras National Seashore, North Carolina. J Coast Res. 2008; 24(6); 1450–1458. 10.2112/07-0856.1

[25] Pombo M, Turra A. Issues to be considered in counting burrows as a measure of Atlantic ghost crab populations, an important bioindicator of sandy beaches. PLoS ONE. 2013; 8(12): e83792 10.1371/journal.pone.0083792 24376748 PMC3871685

[26] Aheto D, Asare C, Mensah E, Aggrey-Fynn J. Rapid assessment of anthropogenic impacts on exposed sandy beaches in Ghana using ghost crabs (*Ocypode* spp.) as ecological indicators. MEJS. 2011; 3(2): 93–103.

[27] Jonah FE, Agbo NW, Agbet W, Adjei-Boateng D, Shimba MJ. The ecological effects of beach sand mining in Ghana using ghost crabs (*Ocypode species*) as biological indicators. Ocean Coast Manag. 2015; 112: 18–24.

[28] Lucrezi S, Saayman M, van der Merwe P. Impact of off-road vehicles (ORVs) on ghost crabs of sandy beaches with traffic restrictions: A case study of Sodwana Bay, South Africa. Envrion Manag. 2014; 53: 520–533. 10.1007/s00267-013-0223-5 24370998

[29] Lucrezi S, Schlacher TA, Robinson W. Human disturbance as a cause of bias in ecological indicators for sandy beaches: experimental evidence for the effects of human trampling on ghost crabs (*Ocypode* spp.). Ecol Indic. 2009; 9 (5): 913–921. 10.1016/j.ecolind.2008.10.013

[30] Schlacher TA, Lucrezi S. Experimental evidence that vehicle traffic changes burrow architecture and reduces population density of ghost crabs on sandy beaches. Vie Milieu- Life Environ. 2010; 60: 313–320.

[31] Lucrezi S, Schlacher TA., Walker S. Monitoring human impacts on sandy shore ecosystems: a test of ghost crabs (Ocypode spp.) as biological indicators on an urban beach. Environ Monit Assess. 2009; 152: 413–424. 10.1007/s10661-008-0326-2 18563608

[32] Kana TW. Beach erosion in South Carolina. South Carolina Sea Grant Consortium. 1988.

[33] Gül MR, Griffen BD. A reliable bioindicator of anthropogenic impact on the coast of South Carolina. Southeast Nat. 2018; 17(2): 357–364.

[34] Wolcott TG. Ecological role of ghost crabs, *Ocypode quadrata* (Fabricius) on an ocean beach: scavengers or predators? J Exp Mar Biol Ecol. 1978; 31(1): 67–82.

[35] R core team. R: A language and environment for statistical computing R Foundation for Statistical Computing, Vienna, Austria 2017.

[36] Chan BKK, Chan KKY, Leung PCM. Burrow architecture of ghost crab *Ocypode ceratophthalma* on a sandy shore in Hong Kong. Hydrobiologia. 2006; 560(1): 43–49. 10.1007/s10750-005-1088-2

[37] Folk RL. Petrology of sedimentary rocks. Hemphill Publishing Company. 1980.

[38] Trivedi JN, Vachhrajani KD. On burrow morphology of the ghost crab *Ocypode ceratophthalmus* (Decapoda;Brachyura: Ocypodidae) from sandy shore of Gujarat, India. International J Mar Sci. 2016; 6(15): 1–10. 10.5376/ijms.2016.06.0015

[39] Packard GC, Boardman TJ. The use of percentages and size-specific indices to normalize physiological data for variation in body size: wasted time, wasted effort? Comp Biochem Physiol. 1999; 122: 37–44. 10.1016/S1095-6433(98)10170-8

[40] Bartoń K. MuMIn: Multi-Model Inference. R package version 1.40.4.2017.

[41] Burnham KP, Anderson DR. Model selection and inference: a practical information-theoretic approach. Springer-Verlag. 2002.

[42] Dixon RW, Peters SL, Townsend CG. Burrowing preferences of Atlantic ghost crab, *Ocypode quadrata*, in relation to sand compaction in Padre Island National Seashore, Texas. Physical Geogr. 2015; 36(3): 188–201. 10.1080/02723646.2015.1033182

[43] Pombo M, de Oliveira AL, Xavier LY, Siegle E, Turra A. Natural drivers of distribution of ghost crabs *Ocypode quadrata* and the implications of estimates from burrows. Mar Ecol Prog Ser. 2017; 565: 131–147. 10.3354/meps11991

[44] de Souza GN, Oliveira CAG, Tardem AS. Counting and measuring ghost crab burrows as a way to assess the environmental quality of beaches. Ocean Coast Manag. 2017; 140: 1–10. 10.1016/j.ocecoaman.2017.02.007

[45] Burrows M, Hoyle G. The mechanism of rapid running in the ghost crab, *Ocypode ceratophthalma*. J Exp Biol. 1973; 58: 327–349.

[46] Blickhan R, Full RJ. Locomotion energetics of the ghost crab: II. Mechanics of the center of mass during walking and running. J Exp Biol. 1987; 130:155–174.

[47] Silva WTAF, Calado TCS. Number of ghost crab burrows does not correspond to population size. Cent Eur J Biol. 2013; 8(9): 843–847. 10.2478/s11535-013-0208-7

[48] Fisher J, Tevesz M. Within-habitat spatial patterns of *Ocypode qudrata* (Fabricius) (Decapoda Brachyura). Crustaceana. 1979; 5:31–36.

[49] Wolcott TG. Uptake of soil capillary water by ghost crabs. Nature. 1976; 264: 756–757. 10.1038/264756a0

[50] Milne LJ, Milne MJ. Notes on the behavior of the ghost crab. Amer Nat. 1941; 80:362–380.

[51] Rodrigues E, Freitas R, Delgado N de C, Soares-Gomes A. Distribution patterns of the ghost crab *Ocypode cursor* on sandy beaches of a tropical island in the Cabo Verde archipelago, Eastern Central Atlantic. Afr J Mar Sci. 2016; 38(2): 181–188. 10.2989/1814232X.2016.1176602

[52] Chakrabarti A. Burrow patterns of *Ocypode ceratophthalma* (Pallas)and their environmental significance. J Paleontol. 1981; 55: 431–441.

[53] Silva WTAF, Calado TCS. Burrow architectural types of Atlantic ghost crab, *Ocypode quadrata* (Fabricius, 1787) (Brachyura: Ocypodidae), in Brazil. bioRxiv. 2014 10.1101/006098

[54] de Oliveira CAG, Souza GN, Soares-Gomes A. Measuring burrows as a feasible non-destructive method for studying the population dynamics of ghost crabs. Mar Biodiv. 2016; 46:809–817. 10.1007/s12526-015-0436-3

[55] Schlacher TA, Lucrezi S, Peterson CH, Connoly RD, Olds AD, Althaus F, et al Estimating animal populations and body sizes from burrows: Marine ecologists have their heads buried in the sand. J Sea Res. 2016; 112: 55–64. 10.1016/j.seares.2016.04.001

[56] Suciu MC, Tavares DC, Zalmon IR. Comparative evaluation of crustaceans as bioindicators of human impact on Brazilian sandy beaches. J Crustacean Biol. 2018; ruy027. 10.1093/jcbiol/ruy027

[57] Defeo O, Rueda M. Spatial structure, sampling design and abundance estimates in sandy beach macroinfauna: some warnings and new perspectives. Mar Biol. 2002; 140(6): 1215–1225. 10.1007/s00227-002-0783-z

